# Antiangiogeneic Strategies in Mesothelioma

**DOI:** 10.3389/fonc.2020.00126

**Published:** 2020-02-18

**Authors:** Anna K. Nowak, Solenn Brosseau, Alistair Cook, Gérard Zalcman

**Affiliations:** ^1^National Centre for Asbestos Related Diseases, University of Western Australia, Crawley, WA, Australia; ^2^Medical School, University of Western Australia, Crawley, WA, Australia; ^3^Institute for Respiratory Health, University of Western Australia, Crawley, WA, Australia; ^4^Department of Medical Oncology, Sir Charles Gairdner Hospital, Nedlands, WA, Australia; ^5^Thoracic Oncology Department & CIC1425-CLIP2 Early Phase Cancer Clinical Trials Unit, University Hospital Bichat-Claude Bernard, Medical Faculty, University Paris-Diderot, Paris, France; ^6^U830 INSERM “Cancer Heterogeneity, Plasticity”, Institute Curie Research Centre, Paris, France

**Keywords:** mesothelioma, angiogenesis, hypoxia, bevacizumab, clinical trials

## Abstract

There is a strong rationale for inhibiting angiogenesis in mesothelioma. Vascular endothelial growth factor (VEGF) is an autocrine growth factor in mesothelioma and a potent mitogen for mesothelial cells. Further, the abnormal tumor vasculature promotes raised interstitial pressure and hypoxia, which may be detrimental to both penetration and efficacy of anticancer agents. Antiangiogenic agents have been trialed in mesothelioma for close to two decades, with early phase clinical trials testing vascular targeting agents, the VEGF-A targeting monoclonal antibody bevacizumab, and numerous tyrosine kinase inhibitors, many with multiple targets. None of these have shown efficacy which has warranted further development as single agents in any line of therapy. Whilst a randomized phase II trial combining the multitargeted tyrosine kinase inhibitor nintedanib with platinum/pemetrexed chemotherapy was positive, these results were not confirmed in a subsequent phase III study. The combination of cisplatin and pemetrexed with bevacizumab, in appropriately selected patients, remains the only anti-angiogenic combination showing efficacy in mesothelioma. Extensive efforts to identify biomarkers of response have not yet been successful.

## Introduction

Malignant mesothelioma is an almost uniformly fatal malignancy aetiologically linked to asbestos fiber inhalation, mainly through occupational exposure. Whilst mesothelioma can develop in the peritoneum, tunica vaginalis, and pericardium, the pleura is the primary site in around 90% of cases (1). Most systemic therapy research has been conducted in malignant pleural mesothelioma (MPM), which will be the focus of this review. Whilst some patients presenting with early disease will undergo aggressive surgery and multimodality therapy, most patients present with advanced disease and palliative systemic therapy will be their mainstay of treatment (2).

Systemic therapy for mesothelioma has not yet benefited from the paradigm shift of personalized medicine. The first demonstration of benefit from systemic therapy of mesothelioma was in 2003, with the EMPHACIS study showing a modest improvement in overall survival (OS) for patients receiving cisplatin/pemetrexed, over cisplatin alone (3). The combination of cisplatin with the antifolate raltitrexed showed similar survival benefits but reported later, and is not widely used (4). The first challenge to this standard of care came in 2016, when the MAPS trial reported a further survival benefit for the addition of bevacizumab to cisplatin/pemetrexed (5). As supported by the NCCN and ASCO guidelines, this has changed the standard of care in some, but not all, parts of the world, due to the lack of FDA registration and universal reimbursement. Here, we discuss the history and role of anti-angiogenic strategies in mesothelioma, with an emphasis on clinical trial data and their clinical application.

## Angiogenesis in Mesothelioma

Tumor vasculature is highly abnormal, with tortuous vessels which can be either distended or pruned, and deviate from the orderly morphology in normal tissues (6). This results in heterogeneity of tumor blood flow, with resulting hypoxia. Excessive vascular leakiness and raised interstitial pressure can further compress the abnormal vasculature, and contribute to poor penetration of anticancer agents into tumor. These characteristics have important consequences for tumor biology and treatment.

**Figure 1:**
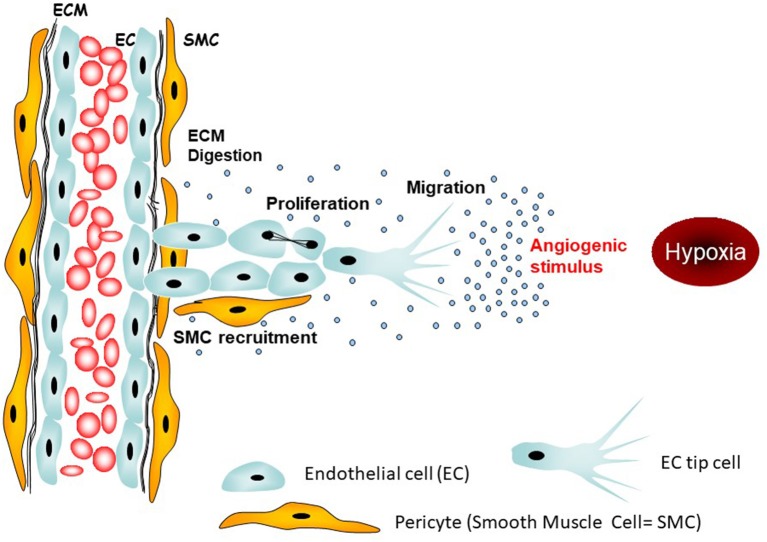
Targetable initial steps of angiogenesis. The main angiogenic stimulus in tumors is hypoxia leading to activation of tip endothelial cells which tract neighboring endothelial cells toward the origin of the stimulus, i.e., the hypoxic region.

Hypoxia is a tumor-promoting state, leading to changes in gene expression that reduce apoptosis (7), enhance receptor tyrosine kinase signaling (8), and promote metastasis (9) and invasion (10), amongst other actions. Hypoxia also has profound immunosuppressive effects and contributes to treatment resistance, most notably to radiotherapy (11). Additionally, hypoxia participates in a feedback cycle which compounds the generation of abnormal tumor vasculature, by upregulating vascular endothelial growth factor (VEGF) and other pro-angiogenic molecules (Figure 1).

Hypoxic conditions lead to HIF-1α and HIF-2 transcription factor stabilization and activation, which in turn control VEGF mRNA production (12). VEGF can also be synthesized in response to nitric oxide (NO) production by the specific endothelial NO-synthase (eNOS) (13). The most extensively studied member of the VEGF family is VEGF-A, secretion of which can be up-regulated in tumor, including mesothelioma, primarily in response to hypoxic stimulus. VEGF-A exists as more than 20 splice isoforms, ranging from 121 to 206 kDa molecular weight; the VEFG_165_ isoform is the most abundant tissue variant. Type B, C, D, E, or F members of the VEGF family have been less comprehensively studied. VEGFs are potent mitogen and survival factors for endothelial cells, signaling through binding to the two receptors, Flt-1 (VEGFR-1) and KDR (VEGFR-2). Activation of VEGFR-2 leads to auto-phosphorylation and downstream signaling through various pathways, such as phosphatidylinositol 3′-OH kinase/Akt. In pleural mesothelioma, VEGF also acts as a powerful mitogen for mesothelial cells themselves. Indeed, mesothelial cell lines secrete VEGF-A and VEGF-C and express both VEGF receptors Flt-1 (VEGF-R1) and KDR (VEGFR-2) (14–16). Thus, VEGF signaling can induce mesothelial cell growth in an autocrine fashion (16–18). This may explain why mesothelioma cells show exquisite sensitivity to anti-VEGF agents, in addition to the more canonical role of such agents in inhibiting neo-angiogenesis.

**Figure 2:**
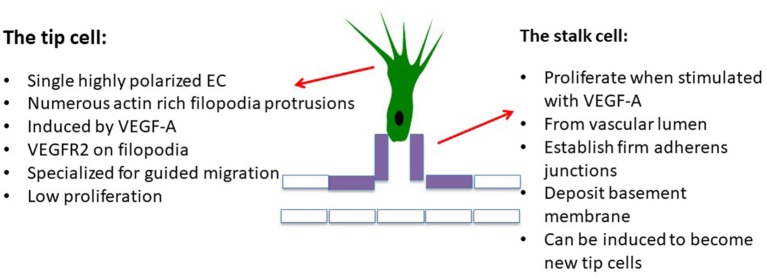
Initial steps, the cell actors: the endothelial tip cell is the first endothelial cell reached by hypoxia-induced stimuli which differentiates into a polarized migrating cell, inhibiting the differentiation of neighbor cells, called “stalk cells,” which passively follow the tip cell, attracting the cell monolayer in which cells adhere to each other via adherens junctions containing VE-cadherin. Stalk cells are still able to proliferate. VEGF-targeting agents are active on both tip and stalk cells, inhibiting both endothelial cell migration and proliferation.

Other growth factors can also regulate migration, survival, and differentiation of endothelial cells, contributing to new vessel development. Factors from the large fibroblast growth factor (FGF) family (aFGFs and bFGGFs) (19, 20) are secreted by both stromal fibroblasts (including pericytes that stabilize new vessels) and tumor cells acting on the FGF receptor (FGFR) family (21). Tumor-associated macrophages, plus endothelial cells, express Tie receptors 1 and 2 for angiopoietins. Angiopoeitins are secreted by endothelial cells and pericytes, and are involved in endothelial cell migration via the process of endothelial tube formation. In addition, vascular cells express Ephrin B2 and B4 [found in mesothelioma (22)] from the ephrin family of tyrosine kinase trans-membrane receptors. These are localized in filopodia of tip endothelial cells that generate vascular spouts during vessel growth and formation (Figure 2). Other proteins expressed by endothelial cells or mesothelial tumor cells, such as TGFβ, EGF, angiogenin, IL-8, and platelet-derived growth factor (PDGF) could also contribute directly or indirectly to endothelial proliferation (23), migration, vessel formation, and stabilization. This complex process may be finely regulated by natural anti-angiogenic proteins such as thrombospondin (24), angiostatin, endostatin, and/or vasostatin (24, 25); these are mainly stocked in the extra-cellular stromal matrix as inactive precursors, and activated by proteolytic cleavage upon activation of matrix metalloproteinases (MMPs). Hence, angiogenesis was a clear rational target in mesothelioma.

## Modulating Angiogenesis in Mesothelioma

Several targeted anti-angiogenic strategies have been used to treat various cancer types: anti-VEGF antibodies i.e., bevacizumab; various tyrosine kinase inhibitors; and other small-molecule inhibitors. Results of trials in mesothelioma have been mixed, as described below.

### Bevacizumab

#### Background on Bevacizumab

Bevacizumab is an anti-VEGF recombinant humanized IgG1 antibody derived from the murine monoclonal antibody A4.6.1 (26). Bevacizumab neutralizes all isoforms of human VEGF, hampering the ability of VEGF to bind to VEGF receptors on the surface of endothelial or mesothelial cells, and inhibiting VEGF-induced proliferation of endothelial cells *in vitro* (26).

**Figure 3:**
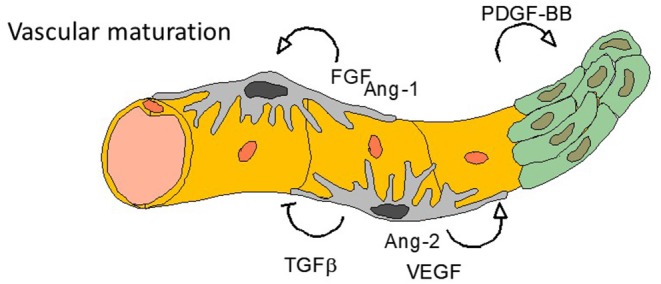
Final steps—vasculogenesis. Tumor angiogenesis is characterized by an anarchic vasculogenesis with immature vessel structure. One of the effect of anti-angiogenic treatments (especially targeting VEGF or VEGFR) is to normalize the vessel cell architecture: all other targetable growth factors listed here are involved in such normalization process.

Preclinical inhibition of VEGF signaling by MAb also decreased tumor vascular permeability in human xenografts implanted into mice (27). These changes, linked to vascular network normalization (Figure 3), are thought to explain the antitumor effects of VEGF inhibitors which can inhibit tumor growth (28) and control micro-metastatic disease in tumor xenografts (29–32). Furthermore, an orthotopic murine xenograft mesothelioma model demonstrated synergy between pemetrexed and bevacizumab compared to the either treatment alone (33). In human studies, bevacizumab has a half-life of around 20 days, and is dosed by weight, 3-weekly, reaching steady state in around 100 days (34).

To our best knowledge there are not preclinical or clinical data about the topical use of bevacizumad, infused directly in the pleural space, although it could theoretically increase mesothelial permeability and help chemotherapy diffusion and efficacy. Bevacizumab was the first anti-angiogenic molecule to be approved by the FDA in 2006, in combination with first-line platinum-based chemotherapy for metastatic non-squamous non-small cell lung cancer (NSCLC). Throughout the last decade, several anti-angiogenic agents have been assessed but none significantly improved survival outcomes, with the exception of nintedanib and ramucirumab in second-line therapy of NSCLC. Nevertheless, as they demonstrated only modest improvement, this did not convince some European countries to fund their reimbursement despite European Medicines Agency approval.

#### Bevacizumab Toxicities

Bevacizumab is generally well-tolerated. Adverse events ≥Gr3 include thromboembolism, hypertension, bleeding, proteinuria, and pulmonary hemorrhage. Meta-analyses demonstrate a bleeding risk of 0.7–0.9%, varying from grade 1–2 (epistaxis) to fatal hemorrhage events like haemoptysis, gastrointestinal bleeding, hematemesis, and cerebral hemorrhage (35–38), similar to reported in MPM (5). The risk of major bleeding in patients with advanced solid tumors is around 2.8% (95% CI 2.1–3.6) (35). Higher risks are observed in patients with NSCLC (RR 3.41, 95% CI 1.68–6.91), renal cell carcinoma (RR 6.37, 95% CI 1.43–28.33), and colorectal cancer (RR 9.11, 95% CI 1.70–48.79) who were receiving bevacizumab 5 mg/kg per week. Use of bevacizumab in squamous cell lung cancer is associated with a high incidence of significant pulmonary hemorrhage, linked to the central location of these tumors, and is currently contraindicated. An increased risk of arterial thromboembolism is also described with anti-angiogenesis therapy (39) while the risk of venous thromboembolism remains controversial with a meta-analysis suggesting no statistically significant increase for bevacizumab compared with control groups (10.9 vs. 9.8%, *p* = 0,13) (40).

As VEGF plays a key role in the maintenance of vascular homeostasis via the NO pathway, VEGF signaling inhibition is associated with arterial vasoconstriction and hypertension. In a large meta-analysis, the incidence of all-grade hypertension was significantly increased at 25.4% of cases (41, 42).

The incidence of proteinuria in patients treated with bevacizumab is 21–63%, but grade 3–4 proteinuria (>3.5 g of protein/24 h, or nephrotic syndrome) occurs in only 1–3% of cases (43). The combination of bevacizumab with chemotherapy significantly increasing the risk for high-grade proteinuria and nephrotic syndrome (43). Few studies *in vivo* have demonstrated that VEGF plays a major role in endothelial development and in repair of glomerular endothelial injury (44).

Bevacizumab is also associated with impaired wound healing (45), likely due to the critical role of VEGF in this process. Whilst the half-life of plasma bevacizumab is 20 days, its tissue half-life is 6 weeks, hence a minimum of 28 days (preferably 6–8 weeks) should elapse between major surgery and the previous dose of bevacizumab (46). Gastrointestinal perforation (GIP) and fistula formation are infrequent but potentially fatal (47).

#### Clinical Trials of Bevacizumab in Malignant Pleural Mesothelioma

The main results of the phase 2 trials assessing bevacizumab in mesothelioma patients are presented in **Table 2**. Jackman et al. evaluated bevacizumab with erlotinib in patients who had previously received chemotherapy (48). In this phase II, multicenter open-label study, 24 patients received erlotinib 150 mg daily and bevacizumab 15 mg/kg every 21 days. The trial did not achieve its primary endpoint and was discontinued.

The first multicenter, double-blind, placebo-controlled, randomized phase II trial of gemcitabine/cisplatin plus bevacizumab in 108 patients with previously untreated and unresectable mesothelioma was published in 2012 (49). Patients received gemcitabine (1,250 mg/m^2^ days 1 and 8 every 21 days), cisplatin (75 mg/m^2^ every 21 days), and either bevacizumab (15 mg/kg) or placebo every 21 days for six cycles, then bevacizumab or placebo every 21 days until progression. The addition of bevacizumab did not significantly improve progression-free survival PFS (6.9 vs. 6 months, *p* = 0.88) or OS (15.6 vs. 14.7 months, *p* = 0.91). There were no significant differences in toxicity. Besides a probably underpowered phase 2 trial, Kindler et al. attributed this disappointing result to a possible negative interaction between gemcitabine and bevacizumab. As shown in preclinical studies, gemcitabine does not mobilize endothelial cell progenitors or increase angiogenesis to the degree observed with taxanes. Another reason may be an unbalanced use of second-line pemetrexed, which was good activity in the second-line setting in patients who have not previously received this drug.

A third phase II study evaluated bevacizumab with carboplatin/pemetrexed as first-line therapy in MPM (50). Patients received pemetrexed 500 mg/m^2^ with carboplatin [area under the plasma concentration–time curve (AUC) 5] plus bevacizumab 15 mg/kg every 21 days for six cycles, followed by maintenance bevacizumab (maximum 1 year). This study did not achieve its ambitious endpoint to show a 50% improvement in median PFS compared to pemetrexed/platinum (from 6 to 9 months), although a longer OS and more long-term survivors were observed in the experimental arm with median PFS (primary endpoint) and OS of 6.9 and 15.3 months, respectively. Treatment was generally well-tolerated, but bowel perforation was reported in 4% of patients, with three toxic deaths.

Finally, Dowell et al. evaluated bevacizumab combined with cisplatin/pemetrexed as first-line treatment in 53 patients with advanced, unresectable MPM (51). The primary objective of a 33% improvement in 6-month PFS with addition of bevacizumab was not met. Median PFS and OS were 6.9 and 14.9 months. Importantly, two fatal adverse events (4%) were possibly related to bevacizumab (one cerebrovascular accident and one small bowel obstruction and fistula).

#### The MAPS Trial

The phase II/III Mesothelioma Avastin Cisplatin Pemetrexed Study (MAPS) was initiated to assess the effect on survival of adding bevacizumab to standard of care chemotherapy as first-line treatment (5). In this large, well-powered, multicenter, randomized, controlled open-label trial, adding bevacizumab to pemetrexed/cisplatin improved both PFS and OS survival compared with pemetrexed/cisplatin alone. Four hundred and forty-eight eligible patients were randomized to receive cisplatin/pemetrexed with or without bevacizumab. Only patients with measurable or evaluable lesions (e.g., pleural effusion) who were younger than 76 years were included, with fewer than 10% of performance status 2 patients. It should be emphasized that large biopsies *via* video-assisted thoracoscopic surgery (VATS) were performed in 85% of patients before chemotherapy, explaining the 10% low rate of recurrent pleural effusion, since thoracoscopy led to efficient pleurodesis.

After six cycles of chemotherapy, the bevacizumab group continued 3-weekly maintenance bevacizumab until progression or toxicity. The primary outcome was OS, and patients were stratified by mesothelioma histology, performance status, and smoking history. After a median follow-up of 39.4 months, patients who received bevacizumab demonstrated significant improvement in median PFS [9.2 vs. 7.3 months; adjusted hazard ratio (HR) = 0.61; *P* < 0.0001] and median OS (18.8 vs. 16.1 months; adjusted HR =0.75; *P* = 0.0167).

As expected, the bevacizumab group experienced more toxicities than the standard chemotherapy group (respectively, 71 vs. 62%). More patients treated with bevacizumab stopped treatment due to toxicity (24.3 vs. 13%), but more patients stopped the treatment due to disease progression in the control arm. Zalcman et al. described more grade 3 hypertension (22 vs. 0%), cardiovascular events (29 vs. 1%), and thrombotic events (6 vs. 1%) in the bevacizumab arm. However, these events were manageable, rarely led to treatment interruptions, and no grade 4 events were observed. Patients receiving bevacizumab experienced more hemorrhage, mainly easily manageable grade 1–2 epistaxis. Strikingly, no haemoptysis was reported. However, notably, patients in this trial were younger than 76 years, and a higher risk of bleeding has been reported in older patients. Only 5 (2.3%) arterial thromboembolic grade 3–4 (and no lower grade) events were observed; this rate was not significantly different between groups. There were more venous thromboembolism grade 2–4 events in the bevacizumab than control arm (12 vs. 3, *p* = 0.02) but the incidence of grade 4 events did not differ statistically between groups. The maximum proteinuria grade was 3 in only 3.2% of patients in the bevacizumab group and did not reduce bevacizumab dose-intensity. No GIP was observed and patients with a previous history of gastro-intestinal surgery were carefully screened before inclusion.

Notably all subgroups (by gender, age, Eastern Cooperative Oncology Group (ECOG) status, PS, or histological subtype) derived an OS benefit from bevacizumab. Patients receiving bevacizumab experienced less fatigue at 9 weeks as assessed by the QLQ-LC30 than control patients. Likewise, significantly more control group patients experienced deteriorating scores for general health on the LCSS-meso at 9 weeks than in the bevacizumab group. A longitudinal QoL study confirmed that not only was bevacizumab not associated with global QoL deterioration, it improved functional scores for two dimensions. There was a clinically significant prolongation of deterioration-free survival for pain scores at 2 months, although this was not statistically significant (HR = 0.85, 95% CI [0.69–1.03], *p* = 0.097). Bevacizumab also significantly delayed the time to deterioration for chemotherapy-related peripheral neuropathy (HR = 0.74, 95% CI [0.61–0.91], *p* = 0.004) (52). Despite these appealing positive results, probably because of the registrations of several bevacizumab biosimilars, the Company marketing bevacizumab took the decision not file the drug for mesothelioma patients, considered to represent a too much limited niche to justify the filing investments needed.

### Anti-angiogenic Tyrosine Kinase Inhibitors

#### Anti-angiogenic Tyrosine Kinase Inhibitors as Single Agents

Interest in anti-angiogenesis in mesothelioma was first noted in the late 1990s, when tyrosine kinase inhibitors targeting these pathways became available. Agents including sunitinib, sorafenib, axitinib, cediranib, and others were tested in a series of single-arm phase II clinical trials, predominantly in the second-line setting, with most trials recruiting fewer than 70 participants. The mains results are presented in Table 1 showing objective radiological response rates were mostly below 15% (54, 57, 59–61, 66). None of these agents proceeded to randomized phase III clinical trials. Notably, many of these agents targeted multiple pathways including not only VEGF receptor isoforms, but also several of the PDGF receptors (PDGFR), FLT4, and others. Despite the targeting of multiple tyrosine kinase receptors, these agents failed to generate meaningful anti-tumor activity against mesothelioma.

**Table 1:** Results from clinical trials of single agent anti-angiogenic and vascular targeting agents in mesothelioma.

**Drug**	**Target**	**Study phase**	**Setting**	**No. of patients**	**Response rate**	**Survival (months)**	**References**
Semaxanib	VEGFR, PDGFR	II	2nd line	9	PR 11%: SD NR	PFS NR; OS 12.4	(53)
Vatalanib	VEGF	II	1st line	47	PR 11%; SD 66%	PFS 4.1; OS 10	(54)
Thalidomide	Angiogenesis	II	1st line 2nd line	40	SD > 6 months: 27.5%	PFS NR; OS 7.6	(55)
NGR-h TNF	NGR-h TNF	II	2nd line	57	PR 2%; SD 44%	PFS 2.8; OS 12.1	(56)
Sunitinib	VEGFR, Flt-1, KDR, Flt-4, PDGFR	II	2nd line	53	PR 12%; SD 65%	PFS 3.5; OS: 7	(57)
Sorafenib	VEGFR, PDGFR, Raf-kinase	II	1st line 2nd line	50	PR 6%; SD 54%	PFS 3.6; OS 9.7	(58)
Cediranib	VEGF-2	II	2nd line	54	PR 9%; SD 34%	PFS 2.6; OS 9.5	(59)
Sunitinib	VEGFR, Flt-1, KDR, Flt-4, PDGFR	II	1st line	18	PR 6%; SD 56%	PFS 2.7; OS 6.7	(60)
			2nd line	17	PR 0%; SD 65%	PFS 2.8; OS 8.3	
Cediranib	VEGFR-2	II	2nd line +	50	PR 10%; SD 34%	PFS 1.8; OS 4.4	(61)
B2P2M2: BNC 105	Vascular disrupting agent	II	2nd line +	30	PR 3%; SD 43%	PFS 1.5; OS 8.2	(62)
Sorafenib	VEGFR, PDGFR, Raf-kinase	II	2nd line	53	PR 6%; SD 56%	PFS 5.1; OS 9	(63)
Pazopanib	VEGFR-1,2,3; cKIT; PDGFR	II		34	PR 6%	PFS 4.2; OS 11.5	Clinicaltrials.gov
Vandetanib	VEGFR, EGFR, RET	II		66	PR 0%; SD 0%	PFS 1.4; OS 7.8	Clinicaltrials.gov
NVALT study: Thalidomide maintenance	Angiogenesis	III	Maintenance	222	Th: NR	Th: PFS 3.6; OS 10.6	(64)
					ASC: NR	ASC: PFS 3.5; OS 12.9	
NGR010	NGR-hTNF, Vascular targeting	III	1st line	400	NGR: DCR 61%	NGR: PFS 3.4; OS 8.5	(65)
					Pl: DCR 47%	Pl: PFS 3.0; OS 8.0	

*NR, Not reported for mesothelioma patients; Clinicaltrials.gov, results extracted from clinicaltrials.gov but not published; Th, thalidomide arm; ASC, active supportive care; NGR, NGR-hTNF arm; Pl, placebo arm*.

#### Anti-angiogenic Tyrosine Kinase Inhibitors in Combination

As a number of anti-angiogenic TKIs had demonstrated modest response rates in mesothelioma, some were trialed in combination with cisplatin/pemetrexed chemotherapy, with the hope that inducing vascular normalization would enhance chemotherapy efficacy. Sunitinib, sorafenib, cediranib, and nintedanib were tested in combination with platinum/pemetrexed (summarized in Table 2), and despite the completion of at least two well-conducted randomized trials, none of these agents demonstrated efficacy that will take them into clinical practice. Here, we will describe in more detail the most conclusive clinical trials incorporating these agents.

**Table 2:** Results from combination clinical trials of anti-angiogenic and vascular targeting agents in mesothelioma.

**Drug**	**Combination**	**Target**	**Study phase**	**Setting**	**No. of patients**	**Response rate**	**Survival (months)**	**References**
Thalidomide	Cisplatin	Angiogenesis	P2	1st line	16	PR 14%; SD 55%	PFS NA; OS 11	(67)
	Gemcitabine			2nd line	22	PR 6%; SD 50%	PFS NA; OS 11	
Bevacizumab	Carboplatin Pemetrexed	VEGF	P1/2	1st line	13	PR 33%	PFS 7.8	Clinicaltrials.gov
Bevacizumab	Cisplatin Pemetrexed	VEGF	P2	1st line	53	PR 40%; SD 35%	PFS 6.9; OS 14.8	(51)
Bevacizumab	Cisplatin	VEGF	RP2	1st line	53	PR 25%	PFS 6.9; OS 15.6	(49)
Placebo	Gemcitabine				55	PR 22%	PFS 6.0; OS 14.7	
Bevacizumab	Carboplatin Pemetrexed	VEGF	P2	1st line	76	PR 34%: SD 58%	PFS 6.9; OS 15.3	(50)
Axitinib	Pemetrexed	PDGFR	RP2	1st line	14	PR 36%; SD 43%	PFS 5.8; OS 18.9	(64)
–	Cisplatin	VEGFR-1,2,3; cKIT			11	PR 18%; SD 73%	PFS 8.3; OS 18.5	
Bevacizumab	Cisplatin	VEGF	P2/3	1st line	223	NR	PFS 9.2^*^; OS 18.8^*^	(5)
–	Pemetrexed				225	NR	PFS 7.3; OS 16.1	
Cedirinib	Pemetrexed Cisplatin	VEGF-2	P1	1st line	20	PR 24%; SD 66%	PFS 8.6; OS 16.2	(68)
Nintedanib	Cisplatin	VEGR 1,2,3; SRC; PDGFR; FGFR; ABL-Kinase	RP2	1st line	44	PR 57%	PFS 9.4^*^; OS 18.3	(69)
Placebo	Pemetrexed				43	PR 44%	PFS 5.7; OS 14.2	
Cediranib	Pemetrexed	VEGF-2	RP2	1st line	45	PR 50%	PFS 7.2; OS 10	(70)
Placebo	Cisplatin				47	PR 20%	PFS 5.6; OS 8.5	
Nintedanib	Cisplatin	VEGR 1,2,3; SRC; PDGFR; FGFR; ABL-Kinase	RP3	1st line	229	PR 45%	PFS 6.8; OS 14.4	(71)
Placebo	Pemetrexed				229	PR 43%	PFS 7.0; OS 16.1	

*PR, partial response; SD, stable disease; PFS, progression free survival; OS, overall survival; RP2, Randomized phase II; NR, not reported*.

* *Denotes a result which was statistically significantly superior to the other study arm*.

*Clinicaltrials.gov, results extracted from clinicaltrials.gov but not published*.

#### Nintedanib and the LUME-Meso Clinical Trials

Nintedanib is an oral angiokinase inhibitor which has multiple targets, including VEGFR1-3, FGFR1–3, PDGFRα/β, RET, Abl, FLT3, and Src (72). When a randomized phase II clinical trial in mesothelioma, LUME-Meso II, was initiated, nintedanib had already been demonstrated safe and tolerable in combination with chemotherapy (72) and a positive clinical trial had been completed in combination with docetaxel as second-line treatment for advanced NSCLC of adenocarcinoma histology (73). Preclinical studies suggested potential activity in mesothelioma (74). LUME-Meso II was initiated to assess the efficacy and safety of nintedanib in combination with cisplatin/pemetrexed (69). This study enrolled 87 participants with chemo-naïve unresectable MPM, ECOG performance status 0–1, and non-sarcomatoid disease histology. Patients were randomized 1:1, double blinded, to cisplatin/pemetrexed with nintedanib 200 mg b.d. or placebo, and nintedanib or placebo was subsequently continued as monotherapy until progression. The study primary endpoint was PFS. Results were released after completion of the randomized phase II portion of the study, strongly favoring the nintedanib-containing arm, with a HR for PFS of 0.54 (95% CI, 0.33–0.87; *P* = 0.010). Although underpowered, OS also showed a trend to benefit with addition of nintedanib (HR, 0.77; 95% CI, 0.46–1.29; *P* = 0.32). Benefits appeared most marked in those patients with epithelioid disease, although patients with non-epithelioid disease only comprised 12% of the study population. The combination appeared safe and tolerable, albeit with a higher incidence of grade 3 neutropenia in the combination group.

These promising results triggered the expansion to a subsequent international confirmatory randomized phase III study, the LUME-Meso-III trial. The phase II observation of more apparent benefit in patients with epithelioid histology, although not paired with an explanatory biological rationale, led to this expansion study excluding those with any other histological subtype; other inclusion criteria remained similar. Patients received an identical treatment regimen to the previous study, including maintenance therapy, and PFS was again the primary endpoint, with a secondary endpoint of OS. The study had statistical power to detect a HR of 0.63 favoring the nintedanib arm (75). A total of 458 patients were randomized in a 1:1 ratio. Unfortunately, there was no difference in PFS between the two arms (HR = 1·01; 95% CI: 0·79–1·30; *p* = 0·91) with a median PFS of 6.8 months in the nintedanib arm and 7.0 months in the placebo arm. The HR for OS was 1·12 (95% CI: 0·79–1·58, *p* = 0·538), with a median survival of 14.4 months in the nintedanib arm and 16.1 months in the placebo arm; there were no new adverse safety signals (71).

Results of the double-blind randomized phase II study “NEMO” from the EORTC Lung Cancer Group, assessing Nintedanib as switch maintenance treatment for MPM patients after disease control obtained with first-line pemetrexed/cisplatin doublet, are still awaited for 2021.

#### Cediranib

Two early-phase clinical trials assessed the efficacy of the single agent VEGF-R tyrosine kinase inhibitor cediranib (AZD2171, Astra-Zeneca) in MPM in the second-line setting (59, 61). Cediranib was also more recently tested combined with pemetrexed/cisplatin as frontline therapy in chemo-naive patients in a phase 1 trial and subsequent randomized phase II trial (68, 70).

The phase 2 trial performed by the Southwest Oncology Group (SWOG) enrolled 54 patients (PS = 0–2) with proven MPM, 47 evaluable, after at least one line of platinum-based chemotherapy and measurable lesions by RECIST. Participants received single-agent cediranib 45 mg daily until progression or toxicity (59). Median PFS was 2.6 months (95% CI: 1.74–3.68), and median OS 9.5 months (95% CI: 5.6–10.7), with 1-year survival of 36% (95% CI: 23–50%); subsequent lines of therapy or patient selection could have played a role in OS which would otherwise be considered acceptable in this disease. Six patients ceased treatment due to adverse events attributed to cediranib, and 43/47 patients had a dose reduction.

Modest activity was also reported in a multi-center phase II trial that accrued 51 unresectable, histologically-confirmed pre-treated MPM patients who received cediranib 45 mg daily (61). Due to toxicity, the starting dose was lowered to 30 mg/d after the 15 first patients. Modest ORR and SD rates are reported in Table 2 and the study did not reach its primary endpoint. No responses were observed in patients with sarcomatoid or biphasic histology. Median PFS was only 1.8 months (95% CI: 0.1–14.2 mo.) and median OS 4.4 months (95% CI: 0.9–41.7 mo.), with 15% 1-year survival. The authors concluded that the limited activity and substantial toxicity did not support use of cediranib single-agent therapy for MPM.

The SWOG phase I study reported first-line therapy combination of cediranib (30 mg/d and 20 mg/d cohorts) with cisplatin/pemetrexed for 6 cycles, followed by maintenance cediranib (68). Twenty chemo-naïve patients with unresectable MPM were enrolled (seven in 30 mg/d cohort, 13 in 20 mg/d cohort). Median PFS was 12.8 months (*n* = 17; 95% CI: 6.9–17.2) by RECIST, and 8.6 months (*n* = 19; 95% CI: 6.1–10.9) using modified RECIST. For all patients, the disease control rate at 6 weeks was 90%, and median OS was 16.2 months (95% CI: 10.5–28.7). Therefore, cediranib combined with cisplatin/pemetrexed was considered to have a reasonable toxicity profile and promising preliminary efficacy—leading to the launching of the S0905 phase II trial which has recently reported (70). In this study, 92 patients with MPM (75% epithelioid, 25% biphasic, or sarcomatoid) were randomized in a 1:1 ratio to platinum/pemetrexed with either cediranib or placebo, followed by maintenance cediranib or placebo. The primary endpoint was PFS via RECIST 1.1. Whilst the addition of cediranib numerically improved PFS by RECIST 1.1 (HR 0.71; *p* = 0.062; 7.2 vs. 5.6 months) there was no significant difference in OS (10 vs. 8.5 months HR 0.88, *p* = 0.28). Toxicity was also problematic, with the addition of cediranib associated with more anorexia, dehydration, diarrhea, and weight loss. This combination is unlikely to move further forward.

### Other Miscellaneous Vascular-Targeting and Vascular-Disrupting Agents

Other vascular-targeting agents have also been trialed in mesothelioma, including NGR-hTNF and BNC-105P. NGR-hTNF is comprised of the N terminal of TNF fused with the C terminal of the tumor-homing peptide NGR (asparagine-glycine-arginine). It targets the aminopeptidase N/CD13 which is expressed on solid tumor endothelial cells, blocking development of new blood vessels, and demonstrating anti-tumor activity (76). An initial single agent phase II study in 43 patients with pre-treated mesothelioma showed manageable toxicity, disease control in 44% of patients (one experiencing PR), and a median PFS of 2.8 months in a cohort treated every 3 weeks. A subsequent 14-patient cohort was treated weekly, with 50% stable disease and median PFS of 3.0 months (56). In hindsight, this is consistent with or even lower than the PFS seen in best supportive care and does not indicate significant activity (77). Nevertheless, given that this agent had the potential to improve the activity of chemotherapy through enhancing penetration into tumor, the international randomized phase III NGR015 study was designed to assess the activity of NGR-hTNF or placebo in combination with investigator choice of management in 400 patients with pre-treated mesothelioma. This study used the weekly regimen of NGR-hTNF, and was partnered with any of gemcitabine, vinorelbine, doxorubicin, or best supportive care. The primary endpoint was OS, which was not different between the two groups (median 8.5 months in the NGR-hTNF group vs. 8.0 months in the placebo group) with a non-significant HR of 0.94 (65). Whilst *post-hoc* subgroup analyses suggested some benefit in those with a shorter prior treatment-free interval, it is unlikely that this agent will be further studied in mesothelioma.

The vascular disrupting agent BNC105P is a small-molecule tubulin polymerase inhibitor that is highly potent and selective for tumor blood vessels, and had preclinical and phase I activity in mesothelioma. This agent was investigated in a single-arm phase II clinical trial as second- or third-line treatment. With an ORR of 3% in 30 patients, and a median PFS of 1.5 months, again there was no evidence of activity (62).

## Biomarkers of Anti-angiogenic Agents

Although there has been over a decade of intense investigation, there are still no clear, validated biomarkers which predict the efficacy of bevacizumab or other anti-angiogenics, either in MPM or in other cancers (78). In the MAPS trial, the prognostic or predictive effect of baseline serum VEGF concentrations were assessed by ELISA in the 372/448 (83%) of patients with available samples. The prognostic analysis based on VEGF assessed as a continuous variable showed that high VEGF concentrations were associated with worse PFS and OS. This was confirmed by bootstrap resampling, a smart statistical method for internal validation of biomarkers, VEGF significantly correlating with worse PFS in 891 (89%) of 1,000 theoretical samples generated by bootstrapping, and with OS in 979 (98%) of 1,000 bootstrapped samples, with high optimism corrected concordance index of 0.64 for PFS and 0.65 for OS. Similar results were obtained by dichotomization at the median value as a cut-off. However, the predictive analysis based on VEGF assessed as a continuous variable showed that the interaction between treatment group and VEGF concentration was not significant for PFS (*p* = 0.60) or OS (*p* = 0.99). An exploratory subgroup analysis according to baseline serum VEGF concentration dichotomized at the median value showed that patients with VEGF concentrations below (adjusted HR 0.56 [95% CI 0.41–0.77]; *p* = 0.0004) or above (0.59 [0.44–0.80]; *p* = 0.0007) the median derived similar benefit in PFS from bevacizumab.

In the group with baseline VEGF concentrations below the median, patients receiving bevacizumab derived a 5.2 months longer OS compared to the chemo-only group (median OS 23.7 vs. 18.5, respectively; adjusted HR 0.73 [0.52–1.03]; *p* = 0.07). Similar results were identified in the study of cisplatin/gemcitabine plus bevacizumab (49). In addition, patients with baseline VEGF concentrations above the median value derived a 2.3 month benefit if they received bevacizumab (15.7 vs. 13.4 months; adjusted HR 0.86 [0.63–1.19], *p* = 0.37). To summarize what is to date the largest prospective study of serum VEGF in MPM patients, high serum VEGF concentration was clearly a worse prognostic biomarker. Regardless, patients with either high or low serum VEGF benefited from bevacizumab—resulting in the conclusion that serum VEGF could not accurately predict a survival benefit upon bevacizumab treatment over chemotherapy-alone treatment. Other studies from the MAPS trial assessing biomarkers for their prognostic/predictive values are still to be presented and published, including baseline plasma concentrations of angiogenesis-regulating micro-RNAs, baseline serum amphireguline, VEGFR immunostaining tumor expression, and microvessel density on CD44 staining. However, no analysis of the effect of BAP1 mutations is available in this study and the influence of such molecular alterations on sensitivity to bevacizumab-containing triplet remains unknown.

There was also extensive investigation of angiogenesis-related biomarkers in the phase II LUME-Meso trial which added nintedanib to chemotherapy. Investigators explored a large panel of putative biomarkers including 58 angiogenic factors by multiplex immunoassay, as well as microvessel density on CD31 staining and germline variants of VEGF. When allowance was made for multiple testing, there were no significant associations with treatment outcome (79).

## Why Did Bevacizumab Succeed and Nintedanib Fail?

Bevacizumab and nintedanib both underwent phase 3 studies in MPM using a very similar design, comparing combination with standard pemetrexed-based chemotherapy over the chemotherapy doublet alone. However, the former showed a significant OS advantage whilst the latter unfortunately resulted in a negative trial; their contradictory fates could derive from both biological and methodological causes.

Biologically, nintedanib concentrations of 20–100 nmol/L block VEGFR, with biochemical IC50 concentrations ranging from 13 to 34 nmol/L on the three VEGFR subtypes—resulting in significant inhibition of endothelial cells, pericytes, and smooth muscle cells proliferation (80). However, such concentrations were shown to be insufficient to reduce survival of lung cancer cell lines, needing much higher concentrations of up to 10 μmol/L (81), above the nanomolar concentrations of most TKI inhibitors used in the clinics. Furthermore, nintedanib shows neither any *in vitro* anti-proliferative effect, nor sensitizes lung tumor cells to chemotherapy, whilst only altering *in vivo* tumor growth by decreasing microvessel density, pericyte coverage, and perfusion, resulting in increased tumor hypoxia (82). Thus, these findings support a purely anti-angiogenic effect for nintedanib, which proved insufficient for an anti-tumor effect in malignant mesothelioma. This suggests that beyond anti-angiogenesis, the inhibition of VEGF-VEGFR signaling pathway would likely work in MPM by inhibiting the autocrine cell growth loop, lacking in other cancer cells such as lung or pancreatic cancer, in which inhibition of VEGFR mainly functions *via* anti-angiogenesis. Of course, this hypothesis remains to be experimentally proven; but would explain a fundamental difference between bevacizumab, a high-affinity binding antibody to VEGF, and a TKI, admittedly efficient on endothelial cells at very low concentrations. Indeed, endothelial cells express a high density of VEGF receptors when compared with MPM cells, in which directly inhibiting the growth factor is needed to alter tumor cell survival. Meanwhile a higher dose of TKI would be needed to inhibit the autocrine loop. Possibly both would be required, because of a lower number of VEGF receptors, and a lower affinity of the receptor than the antibody for VEGF. In addition, nintedanib was recently shown to exert direct anti-tumor effect on tumor cells, but only those with oncogene addiction to growth factors receptors targeted by nintedanib, such as PDGFRα, FGFR2, FLT3, or RET (83).

The second possible reason for the difference in results between these phase 3 trials can perhaps also be found in a putative methodological pitfall of the nintedanib trial. The phase 3 trial had slightly different inclusion criteria compared to the positive nintedanib randomized phase 2—specifically, excluding sarcomatoid or biphasic MPM subtypes (15–20% of MPM). The sponsor claimed that the phase 2 study failed to show any effect in this subpopulation, contrary to the effect observed in the epithelioid subtype. Although it is unlikely that restricting the second study to epithelioid-only patients is the only reason for failure, the phase 2 trial was still not powered to detect any OS difference in the sarcomatoid and biphasic subgroup; a negative result cannot exclude an actual effect without sufficient power, while positivity could reflect a real effect or consist of a false positive result. As an example, the randomized bevacizumab phase II trial by Kindler et al. (49) was presented as negative (although the OS in the two arms were promising), whilst the French phase III was positive. Furthermore, in the phase III trial, bevacizumab's advantage in sarcomatoid and biphasic sup-type was at least as strong as in the epithelioid subtype (if not stronger, since the HR was lower)—suggesting that the statistical interpretation by the Nintedanib trial sponsor may have been erroneous, and could have changed the fate of the Nintedanib phase 3 trial. Of course, we will never know the actual reason of such failure for Nintedanib, and we cannot exclude that there was a mix of biological and methodological reasons contributing to the final negative result. Extensive examination of the data, as well as biomarker studies, has failed to identify a subgroup that may derive benefit, or a reason for failure of LUME-Meso-III.

## Current Recommendations For Use Of Anti-angiogenic Strategies in Mesothelioma

Currently both ASCO (84, 85) and NCCN guidelines (86) suggest that a bevacizumab, pemetrexed and platinum triplet can be used as first-line treatment in PS 0–2 patients with mesothelioma not amenable to radical surgery, without cardiovascular contraindications to bevacizumab, provided there is reimbursement. The national French guidelines “AURA-MESOCLIN” also recommend this strategy although, officially, no reimbursement is assured in France. However, taking into account the small patient numbers (around 1,000 per year in France) and a strong consumer lobby group with occupational asbestos exposure, reimbursement has not been difficult to obtain. In other European countries reimbursement is more uncertain. In the USA insurance companies do reimburse bevacizumab; this not the case in the UK, Australia or Canada. The manufactures of bevacizumab have not submitted an FDA filing for this indication, and it is noted that bevacizumab biosimilar agents are becoming available. Whether biosimilar availability may open access to triplet therapy including a VEGF targeting antibody remains to be seen.

Indeed, a key issue for triplet therapy is cost, and the lack of cost-benefit based on the MAPS data. Most costs derive from direct drug cost rather than indirect toxicity costs, which are generally low grade and manageable. Thus, the cost-benefit varies internationally depending on the drug cost and health system structure in each location. Moreover, previous cost-effectiveness studies in NSCLC or colorectal cancer patients treated with bevacizumab reported conflicting results, likely because of health systems differences. Italian, Taiwanese and Korean studies supported cost-effectiveness, while the UK stated that use of bevacizumab could be associated with increased costs. Chinese and US studies were inconclusive, each with both positive and negative studies (87–93). However, a recent cost-effectiveness study from the IMpower 150 trial, using a Markov model, showed improved cost-effectiveness of an atezolizumab, bevacizumab, carboplatin, and paclitaxel (ABCP) combination over bevacizumab, carboplatin, and paclitaxel (BCP) and carboplatin and paclitaxel (CP) in the first-line treatment of patients with metastatic NSCLC (94). It is difficult to directly extrapolate to mesothelioma patients from NSCLC data, since people with mesothelioma are generally older, but conversely have fewer smoking induced comorbidities. Fewer comorbidities may reduce toxicity, which in turn might lower costs. The lower risk of hemorrhagic complications in the MAPS trial than in NSCLC bevacizumab trials supports this hypothesis.

Finally, because of the lack of any positive phase III studies, no anti-angiogenic TKI has reached the market, and their further development remains uncertain unless efficacy in combination with immune checkpoint inhibitors is demonstrated.

## Incorporating Anti-angiogenics into the Next Generation of Clinical Trials

The next generation of clinical trials in mesothelioma will be split into those that do and do not incorporate bevacizumab in the control arm. The US FDA has not mandated the inclusion of bevacizumab in future clinical trials. Not all patients are eligible for bevacizumab, and more liberal inclusion and exclusion criteria can be considered for trials that do not incorporate bevacizumab, potentially accelerating recruitment and broadening applicability. Bevacizumab is not appropriate for neoadjuvant studies due to impact on wound healing. Furthermore, bevacizumab is not routinely available and used in all jurisdictions, with cost limiting availability in Australia, the United Kingdom, and some parts of Europe.

Nevertheless, there is a strong rationale for testing combinations of chemotherapy, bevacizumab, and checkpoint blockade. VEGF favors tumor recruitment of myeloid-derived suppressor cells (MDSCs), which suppress both T-cell and dendritic cell function thus supporting tumor immune escape (95). VEGF also induces vasodilatation and increases inter-endothelial space, thus favoring extravasation of immune cells that could infiltrate tumor tissue (notably regulatory T cells that can inhibit tumor immune responses). Finally, VEGFR stimulation by its ligands can suppress LATS kinase, leading to nuclear translocation of the YAP transcriptional co-activator and its interaction with TEAD transcriptions factors. This complex activates transcription of several genes involved in the immune response, especially CXCL5, CCL2, PD-L1, CXCR4, and TNF. In parallel, YAP-TEAD activation leads to the transcription of genes involved in stemness such as ALDH1A3 and LGR5, potentially increasing tumor aggressiveness. Hence, the consequences of anti-VEGF therapies are to elicit immune responses through increasing T-cell trafficking into tumors (96, 97), reducing MDSC infiltration (98), reducing regulatory T cells (99), and increasing memory phenotype CD8+ and CD4+ T-cells.

Moreover, in NSCLC, combining atezolizumab with bevacizumab and chemotherapy was efficacious in the IMpower150 phase 3 trial comparing a carbo-paclitaxel-atezolizumab-bevacizumab quadruplet to the triplet therapy (minus atezolizumab) in non-SCC patients. Thus, three early-phase clinical trials are on-going looking for proof-of-concept. The PEMBIB phase Ib trial phase accrued 37 patients with MPM in 2nd or 3rd line setting who subsequently received pembrolizumab with the oral VEGFR TKI Nintedanib. There were no concerning safety signals, and efficacy results are awaited. An MD Anderson Cancer Center trial combined atezolizumab (1,200 mg IV) and bevacizumab (15 mg/kg IV, q21 days) in MPM patients in the same setting: 20 patients were accrued and results are still pending. Twenty patients with peritoneal mesothelioma were also recruited on this study, with results due early 2020. One possible driver to increase testing of combinations is the FDA registration of at least two bevacizumab biosimilars, with more to come, potentially leading to a decrease in drug costs of such combinations.

## Conclusions

In conclusion, the addition of bevacizumab to combination chemotherapy remains an important option for selected patients with MPM, but widespread use as a worldwide standard of care is currently limited by registration and reimbursement considerations. No other antiangiogenic has shown benefit in this setting, and use of other agents should be confined to a clinical trial. This will result in the next generation of clinical trials being those that build on a two-drug combination, and those that build on the triplet combination, and may have the unintended effect of reducing the interpretability and applicability of some future studies. Nevertheless, as not all patients, and not all settings, are appropriate for anti-angiogenic therapy, moving forward to study combinations both with and without bevacizumab remains appropriate.

## Author Contributions

All the authors contributed to the manuscript concept, drafting, revision, and final approval.

### Conflict of Interest

AN declares research funding from AstraZeneca and Douglas Pharmaceuticals. AN declares consultant or advisory positions with honoraria with Bayer pharmaceuticals, Roche, Boehringer Ingelheim, Merck Sharp Dohme, Pharmabcine, Trizell, and Atara Biotherapeutics within the past 5 years. AN declares travel funding from Boehringer Ingelheim and AstraZeneca. GZ declares research funding from Roche and Bristol-Myers-Squibbs, received by the French Intergroup, sponsor of the MAPS and MAPS-2 trials, of which GZ was the former president (2011–2015). GZ declares advisory positions with honoraria perceived by the Fondation pour la Recherche de l'Assistance Publique-Hôpitaux de Paris (Research Fundation of Paris Universtity Hospitals) from Roche, Lilly, Pfizer, Bristol-Myers-Squibbs, Merck-Sharp Dohme, Borhingher-Ingelheim, Paredox Therapeutics, Astra-Zeneca, Da Volterra, Inventiva within the past 5 years. GZ declares travel funding from Astra-Zeneca, Roche, Bristol-Myers-Squibbs, Lilly, Pfizer, Abbvie. The remaining authors declare that the research was conducted in the absence of any commercial or financial relationships that could be construed as a potential conflict of interest.
